# Milk yield and kit development of four breeds of rabbit in Ibadan, Nigeria

**DOI:** 10.1186/s40781-017-0151-7

**Published:** 2017-12-04

**Authors:** Olatunji Abubakar Jimoh, Emmanuel Olabisi Ewuola

**Affiliations:** 1 Animal Physiology and Bioclimatology Unit, Department of Animal Science, University of Ibadan, Ibadan, Oyo State Nigeria; 2 Department of Agricultural Technology, Federal Polytechnic Ado Ekiti, Ado Ekiti, Ekiti State Nigeria

**Keywords:** Survival rates, Litter size, Puberty, Lactation changes, rabbit breeds, Mating systems, Milk yield

## Abstract

**Background:**

Rabbit breeding with high performance imported ones would be of benefit for genetic diversity and improvement of performance in domestic rabbit breeds. The rearing of more productive rabbit breeds could be pathway to improve the productivity and reduce the production cost. Maternal nutritional status exert a great influence on reproductive functions of does, which may expand from conception, through gestation and parturition and development of kits to puberty.

**Methods:**

Four breeds of rabbit were evaluated for their parturition, weaning and pubertal differences among the rabbit population in Ibadan, Nigeria. The breed consist of Fauve De Bourgogne (FDB), Chinchilla (CHA), British Spot (BS) and New Zealand White (NZW) rabbits. A total of 60 bucks and 360 does consisting of 15 bucks and 90 does per breed were mated in 6 mating cycles, three each of natural mating and artificial insemination. All does were synchronized for estrus with 20 IU pregnant mare serum gonadotropin 48 h prior to mating. The does after parturition were assessed for milk yield (g) and kit survival rate (%) till weaning, weight changes of kits from birth to puberty. At puberty, the pubertal age (days) and weight (g) of the offspring were assessed.

**Result:**

Results obtained reveals that British Spot doe had highest milk yield among the breeds which significantly increased growth of kit and weight at weaning in British Spot rabbits. Survival rates of Chinchilla kits were significantly (*p* < 0.05) higher than Fauve de Bourgogne, British Spot and New Zealand White kits. Puberty attainment of the rabbits indicates that British spot does and Fauve de Bourgogne bucks are early maturing.

**Conclusion:**

Chinchilla shows high kit survivability and British spot has highest milk yield among the four breeds of rabbit.

## Background

Rearing rabbits for meat is practised in Nigeria but not on commercial scale. Crossbreeds predominate the rabbit population of Nigeria, and degree of crossing cannot be ascertained due to indiscriminate breeding. Study on pure breeds of rabbits under climatic conditions of West Africa is very scanty, partly due to unavailability of the breeds. The importation of domestic animal diversity is a suitable way to improve performance of rabbits by introducing pure exotic breed of rabbits with high growth and reproductive potentials. The rearing of more productive rabbit breeds could be a suitable path to improve the productivity and reduce the production cost. The most important topic in rabbit research is to improve the production taking into account the farmer requirements, animal welfare and habitat [1].

Maternal nutritional status may exert a great influence on reproductive functions of does, which may expand into the time period after conception, involving early embryogenesis, pregnancy and birth [2]. Negative energy balance, especially in young rabbit does, can result in infertility because of the high energy demands for concurrent pregnancy and lactation [3]. Litter size is the most important economic character in rabbit production [4, 5]. With the increase of litter size and decrease of mortality, income becomes more elevated [6]. The genotype of both the mother and fetuses plays a vital role in determining birth weight, while the consequent litter weights basically depend, beside the fetuses’ genotype, on the suckled milk from the dam [5].

Information and comparative studies undertaken on the reproductive performance of different breeds of rabbit are still scarce and further investigations are required [7]. Four breeds of Rabbit (Fauve de Bourgogne, New Zealand White, Chinchilla and British spot) were recently introduced to rabbit population in Ibadan, at the rabbitry unit of teaching and research farm, of the University of Ibadan, Oyo state, Nigeria, as a policy initiative to improve rabbit production in the region. Without the knowledge of the performance of exotic rabbits, planned improvement of rabbit meat in Nigeria will be limited. The study aimed at documenting the breed difference in parturition, weaning and pubertal characteristics of four breeds of rabbit within rabbit population in Ibadan, Nigeria.

## Methods

### Experimental animals, design and management

Four breeds of rabbit consisting of Fauve de Bourgogne, Chinchilla, British Spot and New Zealand White were used for the study. Before commencement of the trial, animals were confirmed to be of good health status, without abnormalities and conform to the breed and/or age group categorization. The research was approved by the institution’s research ethics committee for care and use of animal for research.

Animals were housed individually and allotted randomly into experimental units in a completely randomized design. The animals were fed 5% of their bodyweight, with diets containing crude protein 17.05%, digestible energy 2592.06 Kcal/kg, crude fibre 10.02%, calcium 0.45% and phosphorus 0.21%. Fresh water was made available to the animals always. Other routine and periodic management practices necessary for rabbit production were carried out.

### Mating trial

A total of 60 bucks and 360 does consisting of 15 bucks and 90 does per breed were mated in 6 mating cycles. This consists of both natural mating (3 mating cycles) and artificial insemination (3 mating cycles). All does used were synchronized for estrus with 20 IU Pregnant mare serum gonadotropins (Ningbo Sansheng Pharmaceutical Co., Ltd) 48 h prior to mating. Natural mating was carried out by moving does to buck at ratio 1:3 buck to doe within breed in the early morning 6–8 am without repeated mating.

For artificial insemination, does were inseminated on-farm with semen extended immediately after collection. Gonadotropin releasing hormone (GnRH; 8 μg) (Ningbo Sansheng Pharmaceutical Co., Ltd) was injected at 0.2 ml intramuscularly at the moment of insemination following estrus synchronisation two days prior or 11 days post partum. The female is placed into a restraining box and inseminated with 0.3 ml of semen containing 5million sperm cells.

**At parturition:**

1. Weight of does was taken before mating and at parturition.
2. Gestation Length: this was taken in days as the difference between the date of mating and kindling date.
3. Litter Size at Birth: This is the number of kits the doe kindles at birth.
4. Litter Birth Weight: This is the weight of the kits at birth. Measurement was taken in grams (g), using a digital scale.

**At weaning:**

1. Litter Size at weaning: This was taken by actual count of weaner alive at weaning.
2. Litter Weight at weaning: Weight of litters taken in g at weaning.
3. Weight of the does was taken at weaning
4. Milk Yield (g): this was estimated by the product of 1.18 and weight gain of kit between birth and 21st day [8].
5. Survival Rates: litter size at weaning/ litter size at birth multiply by 100

### Pubertal characteristics of four breeds of rabbit

#### Does pubertal attainment

Onset of puberty in does was assessed as from 12 weeks of age on a daily basis. Does were observed thrice a day, for signs of estrus i.e. 7–10 am, 4-6 pm and 8-10 pm. Detection of estrus was based on three vital signs namely; increased vascularisation and swelling of the vulva, exposition of the rear quarters, arching of the back (lordosis) and frequent micturition.

Other supporting signs are stretching of the ears, rubbing of the chin on the feed or drinking trough, and aggressive restlessness. The combined observation of the three vital signs [9] and other secondary signs particularly towards the end of a one week monitoring period was considered as “intensive heat” and attract an arbitrary score of 5. The manifestation of any two vital signs with/without other signs was interpreted as “less intense heat” and was assigned a score of 3 whereas one vital sign with/without secondary signs was recorded as “mild heat” with a score of 1. Silent heat was observed when only supporting signs of heat were observed without the vital signs. Every silent heat was scored as 0. Intensity of estrus **(%)** was scored as the mean of observed degree of intensity of estrus over possible maximum intensity multiplied by 100.

### Attainment of puberty assessment

This was determined on the basis of at least 2 of the vital signs of estrus i.e. a score of 3. These signs correspond to the ability of the does to submit to mating [10].

### Bucks pubertal attainment

Onset of puberty in bucks was assessed as from the age of 12 weeks at 72 h interval by examining their preputial fluid smeared on a glass slide with a microscope for sperm cells. Pubertal age was taken as the age when 50% of the tested bucks showed the presence of sperm cells in preputial fluid.

Pubertal characteristic of the weaned litter was assessed. Parameters assessed include: age at puberty is measured in days between date of birth and date the animal attain puberty, weight at puberty of both sexes were determined by weighing the animals at date of puberty.

### Statistical analysis

Data obtained in this study was subjected to descriptive statistics and ANOVA to detect significant effects with a confidence level of 95%.

## Results

### Kit growth and milk yield of four breeds of rabbit in Ibadan

The milk yield and kit growth of four breeds of rabbit in Ibadan is presented in Table 1. The initial weight (at mating), gestation length and litter size at birth and weaning of rabbit across the breeds were not significantly (*p* > 0.05) different. Final weight at parturition was significantly (*p* < 0.05) affected by breeds, with Fauve de Bourgogne, Chinchilla and British Spot have similar values and were significantly (*p* < 0.05) higher than New Zealand White does. Average kit weight at birth was significantly (*p* < 0.05) higher in Chinchilla than Fauve de Bourgogne, British Spot and New Zealand White. Doe gestation weight changes was significantly (*p* < 0.05) highest in Chinchilla, Fauve de Bourgogne had significantly (*p* < 0.05) higher value than New Zealand White. Doe weight at weaning of Chinchilla and New Zealand White had significantly (*p* < 0.05) highest values. Lactation weight changes of New Zealand White does was significantly (*p* < 0.05) highest, Fauve de Bourgogne and Chinchilla does had statistically (*p* > 0.05) similar values but were significantly (*p* < 0.05) higher than values obtained for British Spot does. Kit weight at weaning was significantly (*p* < 0.05) highest in British Spot kits while Fauve de Bourgogne and New Zealand White kits had similar values but they were significantly (*p* < 0.05) lower than Chinchilla kits. Milk yield of Chinchilla and British Spot does were not significantly (*p* > 0.05) different and but were significantly (*p* < 0.05) higher than Fauve de Bourgogne and New Zealand White does. Survival rates of Chinchilla kits had significantly (*p* < 0.05) higher values than Fauve de Bourgogne, British Spot and New Zealand White kits, which had similar values.

**Table 1:** Milk yield and kit growth of four exotic breeds of Rabbit in Ibadan

Parameters	Fauve de Bourgogne	Chinchilla	British Spot	New Zealand White	±SEM
Initial doe weight (g)	2265.64	2277.00	2340.27	2201.00	54.62
Final doe weight (g)	2335.94^b^	2492.00^a^	2422.45^a^	2302.36^b^	36.79
Gestation length (days)	31.47	31.55	32.09	31.73	0.17
Average kit weight at birth (g)	47.19^b^	50.95^a^	47.99^b^	45.51^b^	1.95
Litter size (nos of kits)	5.17	5.43	5.89	6.50	0.47
Doe gestation weight change (g)	108.00^b^	187.63^a^	82.18^bc^	62.50^c^	29.13
Litter size at weaning (nos of kits)	3.56	3.80	3.67	4.00	0.35
Weight of doe at weaning (g)	2237.33^c^	2583.50^a^	2401.17^b^	2507.17^a^	54.16
Doe lactation weight change (g)	20.33^b^	45.67^b^	−4.50^c^	196.17^a^	49.71
Average kit weight at weaning (g)	468.46^c^	575.42^b^	609.13^a^	512.32^c^	32.29
Milk yield (g)	253.14^b^	315.80^a^	350.65^a^	277.47^b^	22.61
Survival rate (%)	76.98^b^	87.670^a^	75.10^b^	74.31^b^	5.98

^a, b, c^: means in the same row with different superscripts are significantly (*P* < 0.05) different. SEM: Standard Error of Mean

### Pubertal age of four breeds of rabbit in Ibadan

**Fig. 1:**
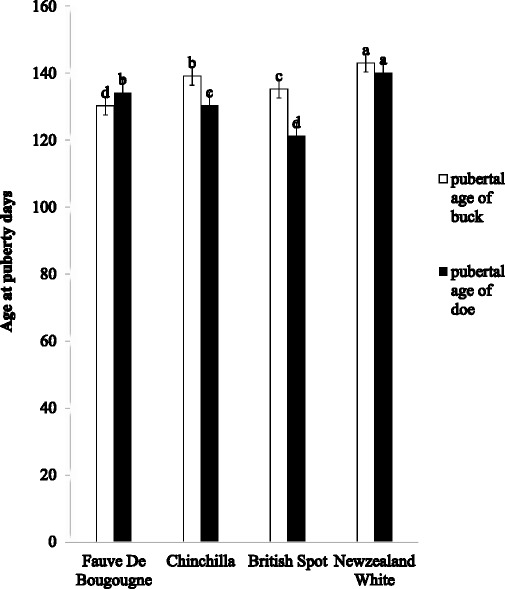
Pubertal age of four exotic breeds of Rabbit in Ibadan

Figure 1 showed the pubertal age of bucks and does of four breeds of rabbit. The pubertal age of doe and buck were significantly (*p* < 0.05) affected by breeds. The pubertal age was significantly (*p* < 0.05) higher in New Zealand White than other breeds. British spot doe and Fauve de Bourgogne bucks had significantly (*p* < 0.05) least pubertal ages. It was observed that pubertal age of bucks were 130.34 days (Fauve de Bourgogne), 139.19 days (Chinchilla), 135.3 days (British Spot) and 143.09 days (New Zealand White). The pubertal age of does ranged from 121.28 days (British Spot) to 140.18 days (New Zealand White).

### Pubertal weight of four exotic breeds of rabbit in Ibadan

**Fig. 2:**
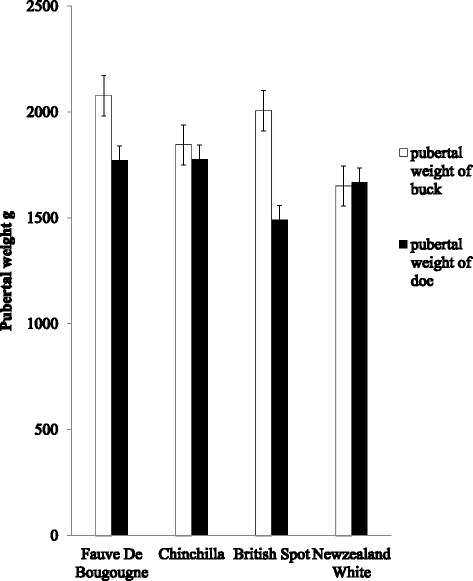
Pubertal weight of four exotic breeds of Rabbit in Ibadan

The pubertal weight of four breeds of rabbit is shown in Fig. 2. Pubertal age of Fauve de Bourgogne, Chinchilla, British Spot and New Zealand White bucks were 2076.75 g, 1844.75 g, 2006 g and 1650.5 g, respectively. The weights of does at puberty across the breeds were 1773.2 g (Fauve de Bourgogne), 1777.2 g (Chinchilla), 1492.17 g (British Spot) and 1669 g (New Zealand White) does.

## Discussion

The breed difference was evident in milk yield and kit development of the breeds of rabbit in Ibadan. Though similar gestation length, litter size at birth and at weaning were recorded. The mothering ability of the breeds was evident. Chinchilla rabbits had higher kit weight at birth resulting from high doe weight gain during gestation. The reverse was the case for New Zealand White rabbits. Conversely, New Zealand White doe had higher weight gain during lactation which compensated for its gestational weight loss. British Spot doe had weight loss during lactation evidently due to milk production judging by its highest milk yield among the breeds which significantly increased growth of kit and weight at weaning in British Spot rabbits. A combination of higher milk yield and lactational weight gain accompanied kit weight at weaning in Chinchilla rabbits. Although Chinchilla kits were heavier than New Zealand White and Fauve de Bourgogne kits at weaning but lower than kits of British Spot. Chinchilla also had the highest kit survival rate. This suggests that high milk yield of British Spot does increased growth of kits to weaning. However, the weight loss during lactation indicates derivation of adipose fat for milk production, implies a longer rebreeding interval, as more time would be required for compensatory growth which is an undesirable trait in rabbit production. A higher plane of nutrition may be required for lactation production function in this breed for optimal milk production and maintenance of dam. Reports demonstrate that maternal nutritional status may exert a great influence on reproductive functions of does, which may expand into the time period after conception, involving early embryogenesis, pregnancy and birth [2]. Similar plane of nutrition was fed to the four exotic breeds, however, weight loss was recorded in British spot despite its high milk yield, and suggests that breed differences could exist in nutritional requirements of the rabbit at different production state.

In line with result obtained in this study are reports by Tag-El-Din [11] and Afifi et al. [12] of non- significant breed differences on litter size. In contrast significant breed differences in litter size between Hyla purebred and crossbred rabbits were reported by Shawer [13] and Akinsola [14]. However, similar range of litter size at birth was reported by Akinsola [14] but lower litter size at weaning in Hyla purebred rabbit in Zaria. This probably is responsible for the higher survival rate in exotic rabbits assessed in this study compared to Hyla rabbit reported by Akinsola [14]. This reflects the superiority in postnatal maternal abilities in terms of milk production, pre-weaning growth and survival, maternal behavior, mothering ability, etc.). Contrary to this result, the values of kit weight at birth and weaning reported by [14] were higher than that observed in this study.

Vaclavovsky et al. [15] reported litter size of 9.49 in Hyla purebred rabbits reared in Slovenia. Chrystosome et al. [16] reported values of 7.29 in France and 6.45 in Benin for Hyla purebred rabbits. Hamouda et al. [17] reported litter size value of 8.50 at first generation with Hyla rabbits in Tunisia. The litter size at birth in this study were also lower than 8.3 reported as average in different rabbit breeds in Australia [18], but closer to 6.30 recorded with New Zealand White rabbits in USA [19]. Litter size at birth obtained from this study were within the range reported by Kabir [20] for pure bred rabbits in Nigeria but lower litter size at weaning compared to values obtained in this study.

The average kit weight at birth obtained in this study, were 47.19, 50.95, 47.99 and 45.51 in Fauve de Bourgogne, Chinchilla, British Spot and New Zealand White, respectively, compared to other authors; the average weights of kits at birth were 58.1 g and 60.2 g [18] in Australia, 43.7 g in Sudanese rabbits [21] and 62.07 g reported by Karikari et al. [22] in Ghana. Akinsola [14] reported higher weaned kits’ weight than the value recorded in similar Hyla rabbit in Tunisia [17], Egypt [23] and Chrystosome et al. [16] in Benin. Though similar litter size at birth with report of Ghosh et al. [24] but values of weight at birth and weaning and litter size in Soviet Chinchilla and Grey Giant rabbit are contrary to result obtained in this study. Abd El-Ghaffar [25] and El Kelawy [26] reported gestation length of New Zealand White (31.2 days.) and Calilfornian (31.5 d.) breeds. Kumaresan et al. [27] reported that the litter size at birth was 7.35 and 7.89 for New Zealand white and Soviet Chinchilla rabbits respectively while the litter size at weaning was 5.25 and 5.65 respectively in India.

Pubertal attainment of the exotic rabbits in Ibadan ranged from 130 to 143 days for Bucks and 121-140 days for Doe. Fauve de Bourgogne Bucks had highest pubertal weight 2076.75 g while New Zealand white had the least (1650.24 g), Chinchilla does had the highest pubertal weight 1777.20 g while British spot had the least 1492.17 g. Puberty attainment of the exotic rabbits indicates that, British spot does and Fauve de Bourgogne bucks are early maturing. However, bucks of Fauve de Bourgogne attain puberty earlier than does. New Zealand White rabbit has the least pubertal attainment for both doe and buck. However, the weight at puberty was similar among breed in both doe and buck, thus suggesting that pubertal weight was a principal factor in determining age at puberty.

Worthy of note is the pubertal age of British spot rabbits, despite a higher weight at weaning, doe weight at puberty was the least. This could suggest lower growth rate post weaning. This suggests that the plane of nutrition was not adequate for the growth rate following withdrawal from milk. This corroborates the earlier suggestion that, the exotic breed differences could require differing plane of nutrition for particular production function. Range of pubertal age obtained in the exotic breeds is lower than Soviet Chinchilla and Grey Giant rabbits 169.39 and 185.60 days respectively reported by Ghosh et al. [24]. Also female rabbits of New Zealand White breeds were heavier at puberty than their male counterparts, in agreement with Ghosh et al. [24], while exotic Fauve de Bourgogne, Chinchilla and British Spot bucks were heavier than does at puberty, though Chinchilla, British Spot and New Zealand White doe attain puberty earlier than bucks.

## Conclusion

Puberty attainment of the exotic rabbits indicate that, British spot does and Fauve de Bourgogne bucks are early maturing. The four breeds show high breeding potential, British spot has high milk yield and its kits had highest growth rate till weaning, but it recorded the least weight at puberty. This is also coupled with doe weight loss experience by the breed during lactation. This indicates inadequacies in growth and maintenance of the breed at certain production phases. This suggests the need for documentation on nutrient requirements of the four exotic breeds of rabbit at different physiological stages and production phase.

## Abbreviations

BS: British Spot
CHA: Chinchilla
FDB: Fauve De Bourgogne
GnRH: Gonadotropin releasing hormone
IU: International unit
NZW: New Zealand White
SEM: Standard Error of Mean
USA: United States of America
